# In sport and now in medical school: examining students’ well-being and motivations for learning

**DOI:** 10.5116/ijme.59b7.8023

**Published:** 2017-09-22

**Authors:** Oksana Babenko, Amber Mosewich

**Affiliations:** 1 Department of Family Medicine, Faculty of Medicine & Dentistry, University of Alberta, Canada; 2 Faculty of Physical Education and Recreation, University of Alberta, Canada

**Keywords:** Medical students, sport, motivation, well-being, admission

## Abstract

**Objectives:**

To investigate relationships between students’ past level of involvement in physical activity/sport and their motivations for learning (achievement goals) and well-being in medical school. In doing so, we provide evidence to medical programs to inform admission processes and curriculum planning.

**Methods:**

A cross-sectional study was conducted. Out of 640 medical students, 267 completed an online questionnaire with measures of: achievement goals, academic burnout, physical activity/sport involvement, and demographics. Data were analyzed using descriptive and inferential statistics (frequency, mean, standard deviation, chi-square test, Cronbach alpha, Spearman correlation).

**Results:**

Students who had pursued physical activity/sport at higher levels of involvement had lower academic burnout scores and endorsed maladaptive achievement goals to a less degree. Specifically, the level of students’ involvement in physical activity/sport was negatively correlated with academic burnout (r=-0.15, p=0.014) and with achievement goals of performance approach (r=-0.15, p=0.014), performance avoidance (r=-0.21, p=0.001), and mastery avoidance (r=-0.24, p<0.001).

**Conclusions:**

Pursuit of dedicated personal activities such as sport appears to be associated with the desired quality of motivation and well-being of medical students. A school culture that fosters resilience of newly admitted students through extracurricular activities and raises students’ awareness of maladaptive and adaptive achievement goals is likely to be beneficial in addressing academic burnout and improving the mental health of medical students.

## Introduction

Medicine is a highly competitive professional education program. In Canada, applicants are expected to meet stringent academic requirements, including high final grades and scores on the Medical College Admission Test (MCAT). Medical schools may also ask prospective students about their involvement in non-academic activities, including pursuit of dedicated or competitive activities such as sport. In a general student population, for example, physical activity/sport involvement has been found to correlate positively with stress tolerance, good time management, and adaptive coping strategies.^1^^,^^2^ The ultimate goal of medical schools is to select applicants with the highest probability of successful medical training and subsequent career.^3^ Although the emphasis has traditionally been placed on prospective students’ academic achievement due to its well-established association with performance in medical school,^4^ the associations between applicants’ non-academic pursuits and learning outcomes in medical school have yet to be established.

Involvement in sport offers invaluable opportunities for an individual to develop self-discipline, resilience, and motivations that may subsequently influence their functioning in stressful, high-stakes environments such as medical school and eventual medical practice. Recreational pursuit of physical activity/sport provides numerous health benefits and can be an effective way of coping with stress.^2^

Competitive pursuit of sport focuses on further skill development in the context of training and frequent competitions where performance outcome is on display (e.g., winning a race, failing to improve on previous results).^5^ Similar parallels can be drawn in medical school, where the focus is on competency acquisition, with frequent assessments of students’ knowledge and skills. 

Individuals applying to medical schools are academically high-performing and motivated to achieve, many of whom have pursued sport at one (or various) points in their lives. Once in medical school, students find themselves surrounded by other high-achieving peers and face many challenges such as mastering large amounts of material in short periods of time and frequent assessments. How do these high-performing students, who have also pursued sport at varying levels, fare in their achievement goals and well-being in medical school? Specifically, are students focused on their performance relative to their high-achieving peers or are they oriented toward personal mastery and learning? Are students able to respond adaptively to the demands of medical studies? To answer these questions, we draw on Achievement Goal Theory (AGT),^6^^-^^8^ a well-established framework that has been applied in the study of motivation across a wide range of achievement settings (e. g., education, sport, work).

Dedicated to understanding the reasons behind the individual’s drive to achieve competence and performance, AGT proposes that individuals adopt certain subconscious orientations – achievement goals – that influence their experience, behaviour, and coping reactions. Elliot and McGregor’s  2×2 framework^7^ distinguishes four achievement goals: (1) performance approach – the desire to look good and demonstrate competence relative to others; (2) mastery approach – the desire to learn, improve skills, or gain competence for its own sake; (3) performance avoidance – the desire to avoid demonstrating incompetence relative to others; and (4) mastery avoidance – the desire to avoid incompetence (i. e., not doing worse than one has done in the past), often accompanied by feelings of not being able to master all the material and fear of making errors. Some individuals can manifest multiple goals at the same time and, depending on the environment they are exposed to, may change their goals as they progress through their education and career.^9^

Across achievement settings, the extensive literature on achievement goals supports the adaptive nature of mastery approach goals relative to the other three goals.^10^ Mastery approach goals have been linked with enjoyment, deeper learning, satisfaction, stress tolerance, and well-being,^10^^-^^12^ which are desired outcomes in medical education. Performance approach goals are considered less adaptive – although these goals are consistently linked to high achievement, they appear also to relate to unfavourable behaviours (e. g., use of surface learning strategies, cheating).^10^^-^^13^ Avoidance goals are considered maladaptive as they are associated with poor psychological well-being and inadequate coping and learning strategies (e. g., worry, anxiety, procrastination, continued use of ineffective learning strategies).^14^^,^^15^

Previous research in achievement goals has focused on personal characteristics such as gender, age, and cultural/ethnic background when examining the goals of individuals in specific settings (e. g., education, sport, work).^15^^,^^16^  We aim to contribute to the existing literature by specifically focusing on medical students who had pursued physical activity/sport at varying levels of involvement and competition. By doing so, we aim to provide evidence that will inform admission and curriculum committees on the relationships between students’ involvement in physical activity/sport, achievement motivation, and well-being in school. This latter aim is in response to recent calls in medical education for research that extends motivation theory to health professions education.^17^^,^^18^ The importance of this cannot be overstated because it is directly linked to preparing internally motivated and resilient health professionals. As such, using the AGT framework, we examined medical students’ well-being (academic burnout) and motivations for learning (achievement goals), while considering the level of students’ past involvement in physical activity/sport.

## Methods

### Study design and participants

This was a cross-sectional survey study. The total sampling scheme was used in the study. Out of 640 undergraduate medical students enrolled in a Canadian university, 267 participated in the study (a response rate of 42%). Overall, 58% of the participants were female and 96% of the participants were 20-29 years old (62% - 20-24 years; 34% - 25-29 years). The distribution of students by year in medical school in this study was approximately even: 27% in the first year, 27% in the second year, 21% in the third year, and 26% in the fourth year.

### Data collection

The survey that was administered to students included measures of physical activity/sport involvement, achievement goals, and academic burnout.

Students were asked to “Please indicate the highest level of physical activity/sport in which you have participated or competed”, with the following response options provided: 1–none; 2–recreational non-competitive; 3–competed in intramurals/a recreational league; 4–competed against athletes from my city/town/nearby communities; 5–competed against athletes from my or another province/state/territory; and 6–competed against athletes from a country other than my own or as a member of a national team. Students were allowed to choose only one response option.

Baranik, Barron, and Finney’s 2×2 achievement goals instrument^19^ was used in the present study, with some adaptations made to better reflect the nature of medical education. Specifically, the words ‘coworkers’, ‘projects’ and ‘work/job’ were changed to ‘others in my program’, ‘tasks’ and ‘program’, respectively. Students were asked to indicate the level of agreement with each statement using a 7-point Likert-type scale (1 – not at all true of me; 7 – yes, very true of me). In total, 16 statements were used to measure four achievement goals: performance approach, mastery approach, performance avoidance, and mastery avoidance. Reliabilities (Cronbach alpha) of the achievement goal measures in this study were as follows: 0.78 for performance approach, 0.79 for mastery approach, 0.81 for performance avoidance, and 0.42 for mastery avoidance. The total scores on each achievement goal could range from 4 to 28, with higher scores indicating greater endorsement of the goal.

For the purposes of this study, burnout was defined as physical, cognitive, and affective exhaustion and was assessed using the 8-item exhaustion scale of the Oldenburg Burnout Inventory – student version (OLBI-S).^20^ Students were asked to indicate the level of agreement with each statement using a 4-point Likert-type scale (1–strongly disagree; 4–strongly agree). The reliability of the academic burnout measure in this study was 0.77. The total scores could range from 8 to 32, with higher scores indicating higher academic burnout.

### Procedure

Using an online survey, quantitative data were collected in October-November 2016, with three reminders sent to all undergraduate students enrolled in the four-year medical program. At the beginning of the survey, the informed consent form explained the purpose and the nature of the study, highlighting voluntary participation and anonymous data collection. Ethics approval was obtained from the Research Ethics Board at the University of Alberta prior to data collection.

### Analyses

Using SPSS 24.0, frequencies and percentages were used to examine the level of involvement in physical activity/sport among the students in the study. Chi-square tests were performed to examine associations between the level of students’ physical activity/sport involvement and gender, age, and year in medical school. Given the potential for social desirability bias in students’ responses, means, standard deviations (SD), and ranges for all the achievement goals and academic burnout items were examined for variability. For each student, scores on four achievement goals and academic burnout were calculated by adding item-level scores on respective items. Next, means and SDs on the achievement goals and academic burnout scales were computed for the group as a whole and for each level of physical activity/sport involvement. These means were then plotted in a graph to examine possible trends in the data. Spearman correlation coefficients were used to examine relationships between students’ physical activity/sport involvement, achievement goals, and academic burnout. Significance level was set at an alpha level of 0.05.

## Results

Overall, 3% (n=7) of the students reported no involvement in physical activity/sport in the past or currently; 33% (n=89) reported recreational non-competitive involvement in physical activity/sport; 24% (n=63) competed in intramurals or in a recreational league; 10% (n=26) competed against athletes from their city/town/nearby communities; 21% (n=55) competed against athletes from around their own or other province/state/territory; and 10% (n=27) competed against athletes from a country other than their own or as a member of a national team. Results of chi-square tests showed no significant associations between the level of past physical activity/sport involvement and students’ gender (χ^2^=4.5, p=0.48), age (χ^2^=14.9, p=0.45), and year in medical school (χ^2^=18.7, p=0.23). Overall, students tended to be predominantly mastery approach-oriented [mean (SD)=22.4 (3.1)]. The levels of endorsement of the other three achievement goals were lower and around the midpoint of their respective scales: performance approach [mean (SD)=16.9(4.4)]; performance avoidance [mean (SD)=13.9(4.3)]; and mastery avoidance [mean (SD)=16.3 (3.3)]. The overall mean (SD) on academic burnout was 20.7 (3.3), suggesting a moderate level of burnout among the students in this study.

**Figure 1:**
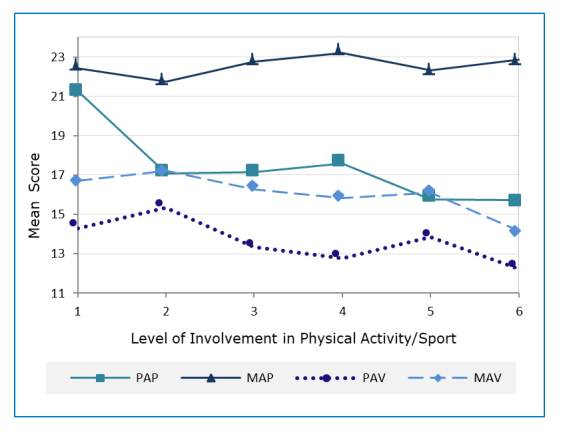
Trends in achievement goals across the levels of physical activity/sport involvement: 1 – none; 2 – recreational non-competitive; 3 – competed in intramurals/a recreational league; 4–competed against athletes from my city/town/nearby communities; 5–competed against athletes from my or another province/state/territory; 6 – competed against athletes from a country other than my own or as a member of a national team. KEY: PAR=Performance Approach; MAP=Mastery Approach; PAV=Performance Avoidance; MAV=Mastery Avoidance

Figure 1 shows trends in achievement goals based on students’ physical activity/sport involvement. An upward trend in mastery approach and downward trends in the other three (maladaptive) achievement goals were observed across the levels of physical activity/sport involvement.

**Table 1:** Correlations of physical activity/sport involvement with achievement goals and academic burnout (n=267)

Variables/Items	Spearman correlation coefficient
Performance Approach (PAP)	-0.15^*^
I prefer to work on tasks where I can show my competence to others	-0.14^*^
I enjoy when others in my program are aware of how well I am doing	-0.20^*^
I like to show that I can perform better than others in my program	-0.11
I try to figure out what it takes to prove my ability to others in my program	-0.07
Mastery Approach (MAP)	0.10
When given a choice, I am willing to select challenging assignments from which I can learn a lot	0.06
I am willing to step out of my comfort zone if it will help develop my competence	0.05
I often look for opportunities to develop new skills and knowledge	0.09
I enjoy difficult tasks in my program where I will learn new skills	0.11
Performance Avoidance (PAV)	-0.21^*^
I prefer to avoid situations in my program where I might perform poorly	-0.11
I am concerned about taking on a task if my performance would reveal that I had low ability	-0.24^*^
Avoiding a show of low ability is more important to me than learning a new skill	-0.17^*^
I would avoid taking on a new task if there was a chance that I would appear incompetent to others	-0.12
Mastery Avoidance (MAV)	-0.24^*^
I just hope I am able to master enough skills so I am competent in my work	-0.03
In my program, I focus on not doing worse than I have done in the past	-0.30^*^
In my program, I often feel that I am unable to master what is necessary to do my work	-0.11
I avoid taking on new tasks when I am not sure I will be able to master them	-0.16^*^
Academic Burnout	-0.15^*^
I can tolerate the pressure of my classes very well (RC)	0.20^*^
When I engage in school work, I usually feel energized (RC)	0.10
When I am studying or doing school work, I often feel emotionally drained	-0.13
Usually I can manage the amount of my school work well (RC)	0.15^*^
After class/school work, I usually feel worn and weary	-0.04
After my class/school work, I have enough energy for my leisure activities (RC)	0.14^*^
There are days when I feel tired before arriving at school	0.01
After classes, I tend to need more time than in the past to relax and feel better	-0.04

*Significant correlations, p<0.05. Physical activity/sport involvement levels: 1–none; 2–recreational non-competitive; 3–competed in intramurals/a recreational league; 4–competed against athletes from my city/town/nearby communities; 5–competed against athletes from my or another province/state/territory; 6– competed against athletes from a country other than my own or as a member of a national team. Achievement goals (PAP, MAP, PAV, MAV) items: 1 – not at all true of me; 7–yes, very true of me. Academic burnout items: 1–strongly disagree; 4–strongly agree. RC: indicates reverse-coded items.

Correlational analyses revealed significant, although small, negative correlations (Table 1) between the level of physical activity/sport involvement and performance approach (r=-0.15, p=0.014), performance avoidance (r=-0.21, p=0.001), and mastery avoidance  (r=-0.24, p<0.001) goals, as well as with academic burnout (r=-0.15, p=0.014), suggesting that with increasing levels of physical activity/sport involvement students tended to endorse maladaptive goals to a lesser degree and experienced less academic burnout in the program. Finally, the correlation between the level of physical activity/sport involvement and mastery approach was positive but did not reach statistical significance (r=0.11, p=0.09).

To better understand the nature of the relationships between physical activity/sport involvement, on the one hand, and achievement goals and academic burnout, on the other, item-level correlations were also examined. As shown in Table 1, the largest significant negative correlations with the level of physical activity/sport involvement were observed for the following items on achievement goals: “I enjoy when others in my program are aware of how well I am doing” (performance approach; r=-0.20, p<0.001); ”I am concerned about taking on a task if my performance would reveal that I had low ability” (performance avoidance; r=-0.24, p<0.001); and “In my program, I focus on not doing worse than I have done in the past” (mastery avoidance; r=-0.30, p<0.001). The negative direction of these correlations suggests that as the level of students’ physical activity/sport involvement increased from none to recreational to competitive, students tended to focus less on how they were doing relative to others in the program. With respect to academic burnout, students with higher levels of past physical activity/sport involvement tended to tolerate the pressure of their classes well, manage the amount of school work well, and have enough energy for their leisure activities after school (Table 1).

## Discussion

Medical education and practice are known for being high-stress and high-stakes environments, which can potentially elicit burnout and poor coping strategies (e. g., alcohol use).^2^^,^^21^^,^^22^ Compared to similar-age individuals in the general population, medical students have substantially higher levels of burnout, stress, and depression.^21^ Searching for solutions, medical school admission committees have started to consider students’ pursuits of dedicated non-academic activities (e. g., sport, music) for their potential beneficial effects on personal and learning outcomes, including motivation and well-being in school. For example, in our medical school, an applicant’s involvement in dedicated non-academic activities is currently weighted at maximum of 30%, depending on the level and duration of such pursuits. In this study, we examined motivations for learning (achievement goals) and well-being (academic burnout) in medical students with varying levels of prior involvement in physical activity/sport.

Using Elliot and McGregor’s 2×2 achievement goals framework,^7^ we observed that medical students in this study were predominantly mastery approach-oriented. Although there was a positive association between mastery approach scores and the level of physical activity/sport involvement, this association did not reach significance. The high level of mastery approach goals among medical students is promising, as these goals are linked to adaptive and beneficial cognitions, behaviours, and learning outcomes. In two recent studies conducted with medical students, mastery approach orientation had the strongest association with general self-efficacy,^23^ frustration tolerance, and perceived psychosocial medical abilities (defined as the level of interest, confidence, clinical abilities, and sensitivity in addressing the psychosocial aspects of patient care).^24^

Compared to mastery approach goals, the endorsement of the other three goals – performance approach, performance avoidance, and mastery avoidance – by medical students in this study was consistently at lower levels. Further, we observed significant negative correlations between the level of past physical activity/sport involvement and these achievement goals, suggesting that with increasing levels of past physical activity/sport involvement the goal orientations toward performance (approach and avoidance) and mastery avoidance in medical school tended to decrease. These results are reassuring in that these achievement goals are known for their maladaptive nature and impact on students’ well-being. Specifically, performance goals relate to undesirable outcomes such as poor study habits, anxiety, and self-handicapping.^25^^,^^26^ Similarly, individuals with mastery avoidance goals are more likely to perceive help-seeking as threatening and experience worry and high stress.^7^^,^^27^ In the present study, the largest negative correlations were observed between students’ level of physical activity/sport involvement and avoidance goals.

Overall, medical students reported a moderate level of academic burnout. However, as the level of past physical activity/sport involvement increased, students reported experiencing lower levels of academic burnout, in particular tolerating the pressures of the school, managing the amount of school work well, and having enough energy for their leisure activities after school. Although the design of the present study does not allow for causal inferences, the significant relationship suggests potential benefit of physical activity/sport involvement in this area and warrants further exploration.

Most notably, only 3% of the students in this study reported no involvement in physical activity/sports at any point in their lives, with the majority of the students having pursued physical activity/sport recreationally. Despite the well-recognized health benefits of physical activity, studies have shown that students’ involvement in such activities and their exercise habits tend to deteriorate upon entry into college.^2^ In a study conducted with medical students in Australia, more than half of the students reported that they did more exercise before the commencement of medical training.^28^ This raises an important question as to what curricular initiatives and learning environments are likely to be effective in helping students maintain their physical activity/sport involvement and in fostering beneficial motivations for learning in medical students. 

### Limitations

There are several inherent limitations in this study. First, this study was based on a cross-sectional survey that provided a preview of the relations between past involvement in physical activity/sport, motivations for learning, and well-being of high-achieving students, specifically students in one medical school. Nevertheless, the students in our medical school are representative of the population of students in medical schools across the country. Second, while we cannot make causal claims, sport involvement does appear to be a positive attribute for students applying to and pursuing medical studies. Third, this study used self-reported data; however, in the context of this study, it reflects the reality in that prospective students also self-report their involvement in extracurricular activities when applying to medical school. Self-report data also poses concerns around social desirability bias. However, students in this study tended to respond using the full range of response options on the measures of achievement goals and academic burnout, yielding evidence against social desirability bias. Fourth, although the response rate in this study was not high but consistent with similar online surveys,^29^it remains unknown whether motivations and well-being of the responding students are similar to those of non-participating students, who, for whatever reasons, did not complete the survey. Finally, the reliability of the mastery avoidance measure was low. Compared to the measures of performance goals, lower reliabilities for mastery goal scales in medical education have also been reported elsewhere.^24^^,^^30^ To address this measurement limitation, we examined both item- and scale-level associations.

### Implications for medical education and future research

This study may serve as a starting point for further research on the impact of students’ pursuit of dedicated extracurricular activities prior to and during medical studies on the quality of students’ motivation and well-being while in school, and once in medical practice. Although we did observe significant correlations between the level of past physical activity/sport involvement and achievement goals and academic burnout, the effect sizes were relatively small. Future studies that take into account the duration of participation and physical activity/sport type (individual vs. team), in addition to the level of involvement, are needed to contribute to our understanding of the attributes that young people can potentially draw upon in their education and professional career. Such attributes can be fostered and promoted. A recent study with otolaryngology residency applicants found that the most predictive variable of future success was an applicant’s prior excellence in team sports.^31^ It stands to reason that if an applicant had a history of success in a team sport, he/she is likely to continue to thrive in teamwork in medical settings.

Beyond the importance of physical activities for optimum health, research indicates that exposure to health-promoting environments in medical school has a strong positive effect on physicians’ attitudes towards preventative medicine and improves their physical activity and patient counseling practices.^32^ Curricular encouragement of physical activity, emphasis on preventative medicine, and provision of extracurricular activities such as physical activity classes and team sports have been shown to be effective health promotion strategies in medical school.^32^

Finally, students’ involvement in other dedicated pursuits such as art and music is another avenue for research. Such exploration would further inform admission processes and guide student supports in medical school. Given the high-stress and high-stakes nature of medical education and practice, it is crucial that we strive to search for the ways in which we can cultivate resilient and successful physicians.

## Conclusions

Medicine is a highly competitive education program and poses many demands for individuals who aspire to health careers. Our findings indicate that the pursuit of dedicated personal activities such as physical activity and sport appears to be associated with the desired quality of motivation and well-being of medical students. A school culture that fosters resilience of newly admitted students through extracurricular activities and raises students’ awareness of the consequences of maladaptive achievement goals is likely to be beneficial in addressing academic burnout and improving the mental health of medical students.

### Acknowledgements

This work was supported by a research grant to the first author from the Social Sciences and Humanities Research Council (SSHRC) of Canada. The authors gratefully acknowledge medical students for their participation in the study and the Faculty of Medicine & Dentistry at the University of Alberta. We thank Lindsey Nadon (MEd Candidate), two anonymous reviewers, and a competitive dedicated cyclist for their thoughtful comments on earlier drafts of this article.

### Conflict of Interest

The authors declare that they have no conflict of interest.
